# Acellular Dermal Matrix (Permacol^®^) for Heterologous Immediate Breast Reconstruction after Skin-Sparing Mastectomy in Patients with Breast Cancer: A Single-Institution Experience and a Review of the Literature

**DOI:** 10.3389/fmed.2016.00072

**Published:** 2017-01-05

**Authors:** Laura Knabben, Gowthami Kanagalingam, Sara Imboden, Andreas R. Günthert

**Affiliations:** ^1^Department of Obstetrics and Gynaecology, University Hospital of Berne, University of Berne, Berne, Switzerland; ^2^Department of Obstetrics and Gynaecology, Cantonal Hospital of Lucerne, Lucerne, Switzerland

**Keywords:** breast cancer, Permacol^®^, acellular dermal matrices, heterologous immediate breast reconstruction, skin-sparing mastectomy

## Abstract

**Objective:**

Skin-sparing mastectomy (SSM) with immediate heterologous reconstruction is a safe oncological option in surgical therapy of early breast cancer. Permacol^®^ is an acellular dermal matrix (ADM) placed between the implant and the skin to improve lower pole projection and implant coverage. The aim of our study was to evaluate the outcome with a focus on patient satisfaction after 6 months and to analyze physical changes of ADM.

**Methods:**

10 patients who underwent SSM with Permacol^®^ were analyzed retrospectively. All patients were followed using a satisfaction questionnaire and an ultrasound evaluation of the tissue thickness of the pectoralis muscle and the Permacol^®^.

**Results:**

No intraoperative complications were observed. One patient required removal of the implant for necrosis after 3 months. Half of the patients underwent secondary corrective surgery. A statistically significant thinning of the pectoralis muscle was observed, compared to the thickening of the Permacol^®^. A majority of the patients were satisfied with the operation, and we found a correlation between lower body mass index and patient satisfaction.

**Conclusion:**

In our small case series Permacol^®^-assisted immediate reconstruction is shown to be an option for selected cases. Physical changes of Permacol^®^ result in a symmetrical coverage of the implant, which may improve cosmetic outcome.

## Introduction

Breast cancer is the most common cancer in women. In Switzerland, about 5,500 women are diagnosed with breast cancer each year (1).

In early breast cancer or in the case of precancerous lesions, skin-sparing mastectomy (SSM) is nowadays an established surgical technique. By conserving the skin envelope and combining this with immediate reconstruction, SSM provides excellent cosmetic results. Furthermore, the oncological safety of SSM has been demonstrated, with recurrence rates being similar to classical mastectomy (2). In the last decades, mastectomy procedures and immediate reconstruction are increasing due to the awareness to provide risk-reducing mastectomy, better cosmetic results, and the patients’ aim (3).

Nevertheless, the complication rate in SSM followed by reconstruction remains an important issue. Adverse outcomes, including skin necrosis, dislocation of the implant, and, capsular contracture, occur in up to 14% of patients (4).

Acellular dermal matrices (ADMs) have been available since the early 1990s. Initially used for abdominal hernia repair and burn treatment, their utilization has expanded to breast reconstruction. Various types of ADMs exist, derived either from human cadaveric dermis (e.g., AlloDerm^®^ and FlexHD^®^) or from porcine dermis (e.g., Permacol^®^ and Strattice^®^). In an elaborate process, cellular components and genetic material are removed; the result is a sterile mesh composed of collagen types I and III and elastin. Histological studies have shown that ADMs become repopulated by host tissue and neovascularized within several days after implantation (5). In a comparative study that looked at the physical characteristics of different ADMs, Permacol^®^ showed the highest values of maximum loads, stiffness, and tensile strength (6).

Acellular dermal matrices are used in one- and two-stage reconstructions. After SSM and placement of the prosthesis, the acellular matrix is sutured to the lower border of the pectoralis muscle to cover the lower pole of the implant. Lower pole projection and the soft tissue coverage are thereby improved. This approach results in a good cosmetic outcome with an acceptable complication rate (7).

Despite a lack of randomized controlled studies to analyze outcomes of breast reconstruction with ADMs, 84.2% of members of the American Society of Plastic Surgeons reported in a survey in 2015 that they used ADMs in breast reconstructions routinely (8).

The aim of the current study is to evaluate the outcome of SSM followed by Permacol^®^-assisted implant-based immediate reconstruction, with a focus on the physical changes of Permacol^®^, cosmetic results, and patient satisfaction.

## Materials and Methods

The first 10 patients to undergo SSM and ADM-assisted immediate breast reconstruction for invasive or *in situ* breast carcinoma, between July 2010 and April 2011 in our certified breast cancer at the University Hospital of Berne, were analyzed in the current study.

Ethical approval from the local ethics committee was obtained, and all patients gave informed consent.

**Figure 1:**
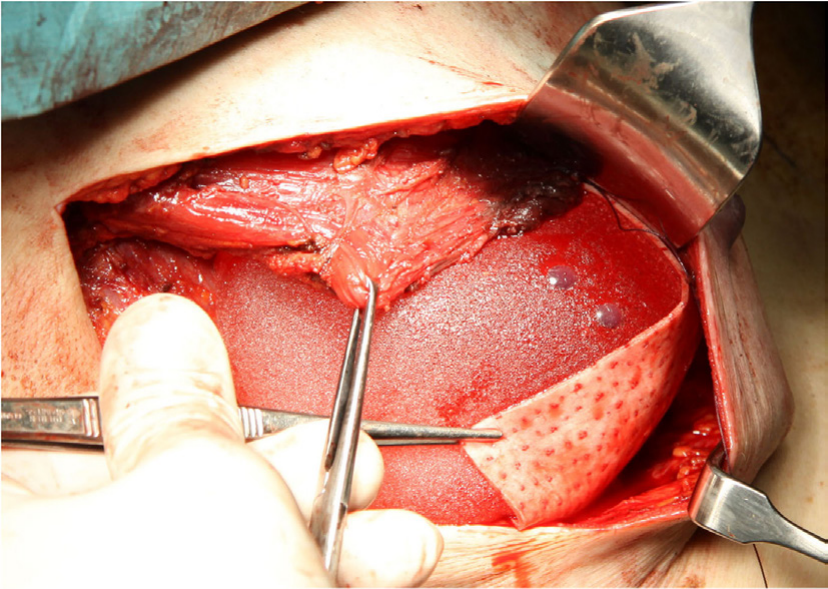
**Intraoperative placement of the implant and Permacol^®^**.

Skin-sparing mastectomy was performed by lateral axillary incision, conserving a maximum of 5 mm of subcutaneous tissue. Resection of breast tissue always included the pectoralis fascia. The decision on nipple areola complex (NAC) preservation was based on the criteria reported by Gerber et al. (9). NAC involvement was excluded by intraoperative frozen section of a retroareolar disk. The implant was placed under the inferior part of the pectoralis muscle. A Permacol^®^ sheet measuring 50 mm × 100 mm × 1.5 mm was sutured to the lower brim of the pectoralis muscle, creating a cup for the implant (Figure 1). The dermis was closed with an absorbable intradermal suture. In all patients at least one drain was placed. Antibiotic therapy was administered until ablation of the drains.

Operation data including duration of surgery, blood loss, implant size, and intra- and postoperative complications were obtained from patient charts.

**Figure 2:**
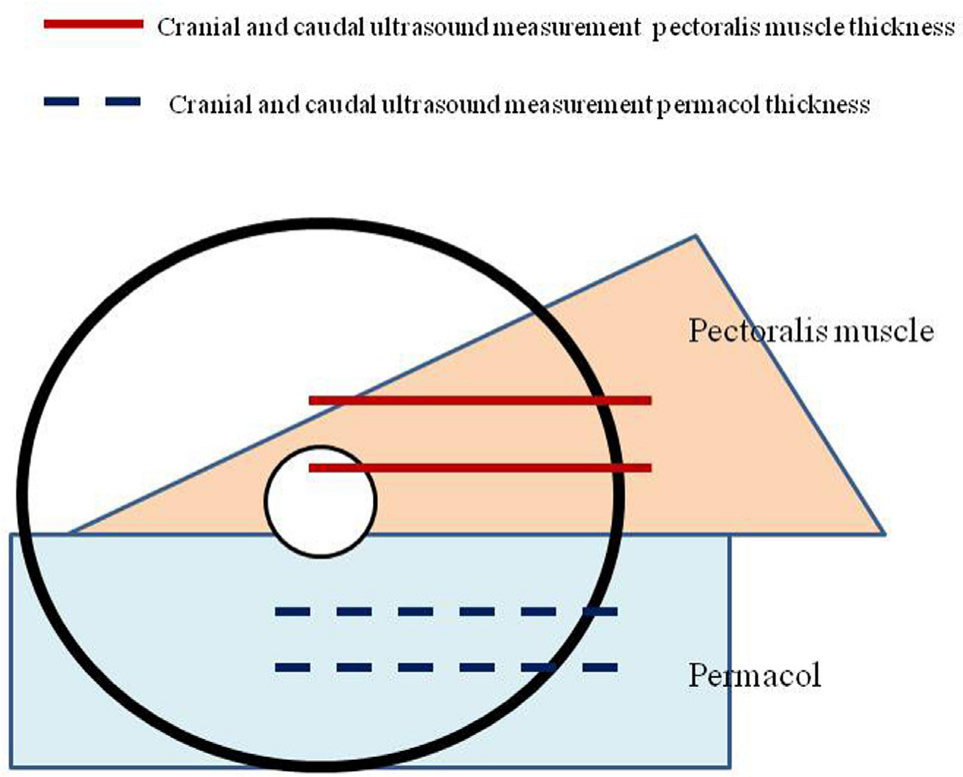
**Defined locations of thickness measurements of the Permacol^®^ and pectoral muscle**.

To evaluate the physical changes of Permacol^®^ after implantation, standardized measurements of the thickness of subcutaneous soft tissue were performed at 1 week and 6 months after operation. The thickness was measured in a horizontal axis at two predefined positions, caudal and cranial of the matrix. These values were then compared to the thickness of the pectoralis muscle at two specific areas (Figure 2).

In the follow-up after 6 months, all patients were questioned using a standard patient-satisfaction questionnaire. The questionnaire consisted of six questions on satisfaction with regard to the outcome and the postoperative experience (Table 1). The answers were scaled using scores from 1 to 5 (1 = strongly agree, 2 = agree, 3 = undecided, 4 = disagree, and 5 = strongly disagree).

**Table 1:** **Postoperative questionnaire**.

1. Is the size and the shape of my breast the same as before the surgery?
2. Are my reconstructed breasts soft?
3. Was corrective surgery necessary?
4. Am I satisfied with the results?
5. If I had the knowledge that I have now after the operation, would I still decide for the breast reconstruction as I did so before having surgery?
6. Would I recommend this surgery to a friend?

Long-term follow-up was evaluated by regular check-ups in our breast center.

Statistical analysis was performed using GraphPad InStat Version 3.10. Quantitative variables were tested by the Student’s *t*-test. Also, *p* < 0.05 was considered statistically significant.

## Results

The indications for SSM with immediate reconstruction were multicentric carcinoma (*n* = 6), diffuse ductal carcinoma *in situ* (DCIS) (*n* = 3), and carcinoma with extensive DCIS (*n* = 1). The mean age of the patients was 50.9 years (37–64 years) at the moment of operation. The average BMI was 21.1 kg/m^2^ (17.6–26.5 kg/m^2^). Two patients had a history of smoking (7.5 and 14 pack years), and one had a past medical history of diabetes mellitus. One patient had a previous tumorectomy with positive resection margins.

Drains were removed after a median of 5.8 days (2–9 days). In all patients, prophylactic antibiotic therapy was administered until ablation of the drains.

No intraoperative complications were observed. The surgical data are summarized in Table 2. Two patients required surgical revision for hematoma, 2 and 4 days after initial surgery. In one patient, the implant had to be removed due to severe skin necrosis 3 months after initial operation; the patient smoked and had previous ipsilateral breast surgery. One patient developed capsule fibrosis after 6 months and received an autologous reconstruction.

**Table 2:** **Surgery data**.

Patient	Blood loss (ml)	Duration (min)	Implant size (mm)
1	200	120	165
2	200	120	295
3	200	120	335
4	400	90	420
5	200	160	390
6	500	120	240
7	10	45	220
8	300	120	180
9	400	120	210
10	200	120	285
Mean	261	113	274

The long-term follow-up with a median of 45.7 months (19–60 months) was uneventful in 5 out of 10 patients. One patient required minor corrective surgery. Three patients developed severe capsular contracture and received implant explanation followed by autologous reconstruction 7, 19, and 26 months after initial surgery. One patient was operated recently in response to suspicion of implant rupture and received reconstruction with a DIEP-Flap.

We also analyzed the physical changes of Permacol^®^. We found a reduction in thickness of the pectoralis muscle from 9.8 to 4.1 mm (cranial) (*p*< = 0.03) and from 8.8 to 4.5 mm (caudal) (*p* < 0.03).

The thickness of Permacol^®^ increased from 1.5 to 1.9 mm (cranial) (*p* = 0.064) and from 1.5 to 2.0 mm (caudal) (*p* = 0.051).

We evaluated the patient’s satisfaction 6 months postoperatively *via* a questionnaire. The majority (*n* = 8) of the patients were satisfied with the similarity of the breast shape pre- and postoperatively. Only one patient noted a considerable change after capsule fibrosis. Most patients were indifferent when responding to the question on whether their reconstructed breast was soft: seven gave a score of 3–4. None of the patients had their implants removed because of infection. Most patients (*n* = 7) were satisfied with the results of the surgery (scores of 1 and 2), with only two patients answering somewhat negatively (scores of 3 and 4). Six patients would do this surgery again. Six patients would recommend this surgery to a friend; only one patient responded that she would absolutely not recommend the surgery.

**Figure 3:**
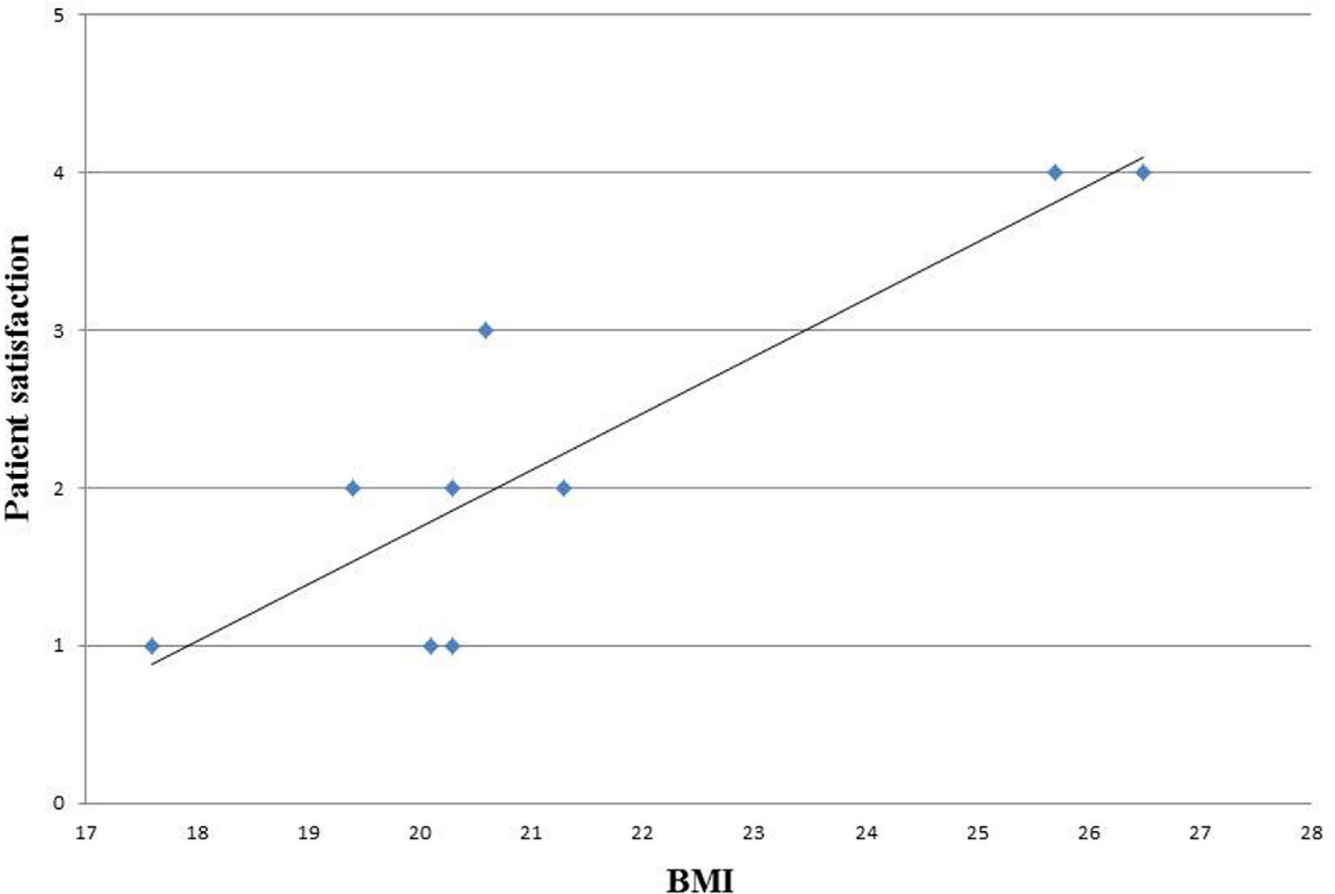
**Correlation between BMI and patient’s satisfaction**.

We analyzed also the correlation between BMI and patients satisfaction and observed that the satisfaction level decreased with increasing BMI (Figure 3).

## Discussion

The major finding of our study is that SSM followed by immediate Permacol^®^-assisted heterologous breast reconstruction is a feasible operation with no intraoperative complications for patients with breast cancer. Furthermore, we could observe a significant increase in the thickness of the Permacol^®^ sheet over the implant and at the same time a significant decrease in the thickness of the pectoralis muscle. This results in a symmetrical surface over the implant.

Although some advantages of Permacol^®^ were observed including the physical changes and lower costs, the disadvantages as low elasticity and the high percentage of required corrective surgery in our cohort finally led us to prefer alternative matrices.

To the best of our knowledge, there are only few studies to date that analyze the physical changes of ADMs *in vivo*. O’Brien examined histological and mechanical results of an explanted piece of Permacol^®^ 2 years after abdominal wall repair; he found an increased breaking strength due to the integration of human collagen and elastin, without any sign of inflammation (10). In 2011, Mulier demonstrated in an experiment on 89 rats that Permacol^®^ maintained thickness and showed greater tensile strength than Strattice^®^, which decreased significantly in thickness starting at 3 months (11). Further studies must be conducted to deepen the understanding of mechanical and physical changes of ADMs *in vivo*.

The use of ADMs in breast reconstruction has become very popular in the last few years. Whereas in 2010, 50% of plastic surgeons reported using ADMs in their daily practice for breast reconstruction, and in 2015, 84.2% of plastic surgeons in the United States reported using it routinely (8, 12). However, the literature indicates that there is a lack of randomized controlled trials evaluating the outcome of ADM-assisted breast reconstruction. The data are poor concerning esthetic results and patient satisfaction.

A recent review and meta-analysis concluded that the use of ADMs appeared to enhance the risk of infection, seroma, and necrosis, but the risk of serious complications and overall mortality might not be increased (13). The rate of capsular contracture seems to be reduced by the use of ADMs (14).

In our study cohort, follow-up showed a need for corrective surgery in about half of the patients. Two patients required revision in the immediate postoperative period, and one patient suffered from necrosis 3 months after the operation. The long-term follow-up revealed that four patients developed capsular contracture, which necessitated implant removal in a mean time of 27 months (7–56). This may be partially due to a learning curve, as we analyzed in this study the first 10 patients operated by ADM-assisted breast reconstruction in our center. It is however a well-known problem in breast reconstructions that follow-up operations are needed to optimize the esthetic outcome. This must be emphasized in preoperative counseling. One important factor in choosing the appropriate reconstructive surgery is patient selection. We noticed a direct correlation between BMI and patient satisfaction. This finding is consistent with other authors’ showing a correlation between higher BMI and wound infection, reoperations, and major complication rates (15, 16). Other risk factors identified are smoking and previous surgery or adjuvant radiotherapy (17).

These aspects might help us identify the“ideal”patients for reconstructive breast surgery with Permacol^®^.

However, the follow-up questionnaire at 6 months postoperatively revealed that the majority were satisfied with the outcome and would recommend the operation to a friend. We are convinced of the importance of preoperative counseling including explanation of potential complications but also of possible benefit of the use of ADMs in breast reconstruction; furthermore, the careful patient selection is crucial.

## Conclusion

In our small case series, the SSM using Permacol^®^ for immediate heterologous breast reconstruction is shown to be an option for selected cases. Physical changes of Permacol^®^ result in a symmetrical coverage of the implant, which may improve cosmetic outcome. We observed an acceptable patient-satisfaction rate. This study further showed good feasibility and no severe direct postoperative complications. A careful patient selection, according to BMI and other risk factors, has to be performed in order to reduce the number of follow-up operations.

## Ethics Statement

This study was carried out in accordance with the recommendations of local ethical committee of Berne with written informed consent from all subjects. All subjects gave written informed consent in accordance with the Declaration of Helsinki. The protocol was approved by the KEK Berne.

## Author Contributions

LK: data analysis and writing of the manuscript. GK: data collection. SI: writing of the manuscript. AG: idea to the project and correction of the manuscript.

## Conflict of Interest Statement

The authors declare that the research was conducted in the absence of any commercial or financial relationships that could be construed as a potential conflict of interest.
